# The Memphis Journal of the Medical Sciences

**Published:** 1890-06

**Authors:** 


### BOOK NOTICES.

The Memphis Journal of the Medical Sciences for April, 1890, is at hand. Among other interesting things is an article upon "Experimental Diabetes Mellitus," translated from the German by Dr. Wm. Krauss.

According to this article Dr. Minkowski has succeeded in producing diabetes in dogs by extirpation of the pancreas. "The entire pancreas had to be removed to cause diabetes, it being immaterial whether the remaining portion was in connection with the duct or not." Ligature of the pancreatic duct would not produce the disease.

From these experiments the disease seems to depend upon the "loss of an as yet unknown specific function of the pancreas in the intermediate metabolism, a function that is absolutely necessary for the destruction of sugar in the organism."

Diabetes produced in this way is the only experimental form analogous to the corresponding disease in man. Coincidently it is found that among post-mortem appearances of diabetes the pancreas appears to be diseased oftener than any other organ, sometimes as high as fifty per cent. of cases. This peculiar influence of the pancreas, the experimenter thinks, "need be only a link in the chain of general glycosic metabolism," though he has no positive proof as yet for his hypothesis. W. M. J.

